# Common Genetic Variation in Circadian Rhythm Genes and Risk of Epithelial Ovarian Cancer (EOC)

**DOI:** 10.23937/2378-3648/1410017

**Published:** 2015-09-15

**Authors:** Heather S.L. Jim, Hui-Yi Lin, Jonathan P. Tyrer, Kate Lawrenson, Joe Dennis, Ganna Chornokur, Zhihua Chen, Ann Y. Chen, Jennifer Permuth-Wey, Katja KH. Aben, Hoda Anton-Culver, Natalia Antonenkova, Fiona Bruinsma, Elisa V. Bandera, Yukie T. Bean, Matthias W. Beckmann, Maria Bisogna, Line Bjorge, Natalia Bogdanova, Louise A. Brinton, Angela Brooks-Wilson, Clareann H. Bunker, Ralf Butzow, Ian G. Campbell, Karen Carty, Jenny Chang-Claude, Linda S. Cook, Daniel W. Cramer, Julie M. Cunningham, Cezary Cybulski, Agnieszka Dansonka-Mieszkowska, Andreas du Bois, Evelyn Despierre, Weiva Sieh, Jennifer A. Doherty, Thilo Dörk, Matthias Dürst, Douglas F. Easton, Diana M. Eccles, Robert P. Edwards, Arif B. Ekici, Peter A. Fasching, Brooke L. Fridley, Yu-Tang Gao, Aleksandra Gentry-Maharaj, Graham G. Giles, Rosalind Glasspool, Marc T. Goodman, Jacek Gronwald, Philipp Harter, Hanis N. Hasmad, Alexander Hein, Florian Heitz, Michelle A.T. Hildebrandt, Peter Hillemanns, Claus K. Hogdall, Estrid Hogdall, Satoyo Hosono, Edwin S. Iversen, Anna Jakubowska, Allan Jensen, Bu-Tian Ji, Beth Y. Karlan, Melissa Kellar, Lambertus A. Kiemeney, Camilla Krakstad, Susanne K. Kjaer, Jolanta Kupryjanczyk, Robert A. Vierkant, Diether Lambrechts, Sandrina Lambrechts, Nhu D. Le, Alice W. Lee, Shashi Lele, Arto Leminen, Jenny Lester, Douglas A. Levine, Dong Liang, Boon Kiong Lim, Jolanta Lissowska, Karen Lu, Jan Lubinski, Lene Lundvall, Leon F.A.G. Massuger, Keitaro Matsuo, Valerie McGuire, John R. McLaughlin, Ian McNeish, Usha Menon, Roger L. Milne, Francesmary Modugno, Lotte Thomsen, Kirsten B. Moysich, Roberta B. Ness, Heli Nevanlinna, Ursula Eilber, Kunle Odunsi, Sara H. Olson, Irene Orlow, Sandra Orsulic, Rachel Palmieri Weber, James Paul, Celeste L. Pearce, Tanja Pejovic, Liisa M. Pelttari, Malcolm C. Pike, Elizabeth M. Poole, Eva Schernhammer, Harvey A. Risch, Barry Rosen, Mary Anne Rossing, Joseph H. Rothstein, Anja Rudolph, Ingo B. Runnebaum, Iwona K. Rzepecka, Helga B. Salvesen, Ira Schwaab, Xiao-Ou Shu, Yurii B. Shvetsov, Nadeem Siddiqui, Honglin Song, Melissa C. Southey, Beata Spiewankiewicz, Lara Sucheston-Campbell, Soo-Hwang Teo, Kathryn L. Terry, Pamela J. Thompson, Ingvild L. Tangen, Shelley S. Tworoger, Anne M. van Altena, Ignace Vergote, Christine S. Walsh, Shan Wang-Gohrke, Nicolas Wentzensen, Alice S. Whittemore, Kristine G. Wicklund, Lynne R. Wilkens, Anna H. Wu, Xifeng Wu, Yin-Ling Woo, Hannah Yang, Wei Zheng, Argyrios Ziogas, Ernest Amankwah, Andrew Berchuck, Georgia Chenevix-Trench on behalf of the AOCS management group ,, Joellen M. Schildkraut, Linda E. Kelemen, Susan J. Ramus, Alvaro N.A. Monteiro, Ellen L. Goode, Steven A. Narod, Simon A. Gayther, Paul D. P. Pharoah, Thomas A. Sellers, Catherine M. Phelan

**Affiliations:** 1 Department of Health Outcomes and Behavior, Moffitt Cancer Center, Tampa, FL, USA; 2 Department of Biostatistics and Bioinformatics, Moffitt Cancer Center, Tampa, FL, USA; 3 Department of Public Health and Primary Care, The Centre for Cancer Epidemiology, University of Cambridge, Strange ways Research Laboratory, Cambridge, UK; 4 Department of Preventive Medicine, Keck School of Medicine, University of Southern California Norris Comprehensive Cancer Center, Los Angeles, CA, USA; 5 Department of Cancer Epidemiology, Division of Population Sciences, Moffitt Cancer Center, Tampa, FL, USA; 6 Radboud University Medical Center, Radboud Institute for Health Sciences, Nijmegen, The Netherlands; 7 Netherlands Comprehensive Cancer Organization, Utrecht, The Netherlands; 8 Genetic Epidemiology Research Institute, UCI Center for Cancer Genetics Research and Prevention, School of Medicine, Department of Epidemiology, University of California Irvine, Irvine, CA, USA; 9 Byelorussian Institute for Oncology and Medical Radiology Aleksandrov N.N., Minsk, Belarus; 10 Cancer Epidemiology Centre, Cancer Council Victoria, Melbourne, Australia; 11 Cancer Prevention and Control, Rutgers Cancer Institute of New Jersey, New Brunswick, NJ, USA; 12 Department of Obstetrics & Gynecology, Oregon Health & Science University, Portland, OR, USA; 13 Knight Cancer Institute, Oregon Health & Science University, Portland, OR, USA; 14 Department of Gynecology and Obstetrics, University Hospital Erlangen, Friedrich-Alexander-University, Erlangen-Nuremberg Comprehensive Cancer Center, Erlangen EMN, Germany; 15 Department of Surgery, Gynecology Service, Memorial Sloan-Kettering Cancer Center, New York, NY, USA; 16 Department of Gynecology and Obstetrics, Haukeland University Hospital, Bergen, Norway; 17 Centre for Cancer Biomarkers, Department of Clinical Medicine, University of Bergen, Bergen, Norway; 18 Gynecology Research Unit, Hannover Medical School, Hannover, Germany; 19 Division of Cancer Epidemiology and Genetics, National Cancer Institute, Bethesda, MD, USA; 20 Canada’s Michael Smith Genome Sciences Centre, BC Cancer Agency, Vancouver, BC, Canada; 21 Department of Biomedical Physiology and Kinesiology, Simon Fraser University, Burnaby, BC Canada; 22 Department of Epidemiology, University of Pittsburgh Graduate School of Public Health, Pittsburgh, PA, USA; 23 Department of Obstetrics and Gynecology, University of Helsinki and Helsinki University Central Hospital, Helsinki, HUS, Finland; 24 Department of Pathology, Helsinki University Central Hospital, Helsinki, HUS, Finland; 25 Cancer Genetics Laboratory, Research Division, Peter MacCallum Cancer Centre, St Andrews Place, East Melbourne, Australia; 26 Department of Pathology, University of Melbourne, Parkville, Victoria, Australia; 27 Sir Peter MacCallum Department of Oncology, University of Melbourne, Parkville, Victoria, Australia; 28 Department of Gynaecological Oncology, Glasgow Royal Infirmary, Glasgow, G31 2ER, UK; 29 CRUK Clinical Trials Unit, The Beatson West of Scotland Cancer Centre, 1053 Great Western Road, Glasgow G12 0YN, UK; 30 German Cancer Research Center (DKFZ), Division of Cancer Epidemiology, Heidelberg, Germany; 31 Division of Epidemiology and Biostatistics, Department of Internal Medicine, University of New Mexico, Albuquerque, NM, USA; 32 Obstetrics and Gynecology Center, Brigham and Women’s Hospital and Harvard Medical School, Boston, MA, USA; 33 Department of Laboratory Medicine and Pathology, Mayo Clinic, Rochester, MN, USA; 34 International Hereditary Cancer Center, Department of Genetics and Pathology, Pomeranian Medical University, Szczecin, Poland; 35 Department of Pathology, The Maria Sklodowska-Curie Memorial Cancer Center and Institute of Oncology, Warsaw, Poland; 36 Department of Gynaecology and Gynaecologic Oncology, Kliniken Essen-Mitte/ Evang. Huyssens-Stiftung/Knappschaft GmbH, Essen, Germany; 37 Department of Gynaecology and Gynaecologic Oncology, Dr. Horst Schmidt Kliniken Wiesbaden, Wiesbaden, Germany; 38 Division of Gynecologic Oncology; Leuven Cancer Institute, University Hospitals Leuven, KU Leuven, Leuven, Belgium; 39 Department of Health Research and Policy-Epidemiology, Stanford University School of Medicine, Stanford, CA, USA; 40 Department of Epidemiology, Geisel School of Medicine, Dartmouth, Hanover, NH, USA; 41 Program in Epidemiology, Division of Public Health Sciences, Fred Hutchinson Cancer Research Center, University of Washington, Seattle, WA, USA; 42 Department of Gynecology, Friedrich Schiller University, Jena, Germany; 43 Department of Oncology, Centre for Cancer Genetic Epidemiology, University of Cambridge, Cambridge, UK; 44 Department of Public Health and Primary Care, Centre for Cancer Genetic Epidemiology, University of Cambridge, Cambridge, UK; 45 Wessex Clinical Genetics Service, Princess Anne Hospital, Southampton, UK; 46 Department of Obstetrics Gynecology/RS, Division of Gynecological Oncology, Ovarian Cancer Center of Excellence, University of Pittsburgh, Pittsburgh, PA, USA; 47 Institute of Human Genetics, University Hospital Erlangen, Friedrich-Alexander-University Erlangen-Nuremberg, Erlangen, Germany; 48 Department of Medicine, Division of Hematology and Oncology, University of California at Los Angeles, David Geffen School of Medicine, Los Angeles, CA, USA; 49 Department of Biostatistics, University of Kansas Medical Center, Kansas City, KS, USA; 50 Department of Epidemiology, Shanghai Cancer Institute, Shanghai, China; 51 Women’s Cancer, UCL EGA Institute for Women’s Health, London, UK; 52 Centre for Epidemiology and Biostatistics, School of Population and Global Health, The University of Melbourne, Melbourne, Australia; 53 Cancer Prevention and Control, Samuel Oschin Comprehensive Cancer Institute, Cedars-Sinai Medical Center, Los Angeles, CA, USA; 54 Department of Biomedical Sciences, Community and Population Health Research Institute, Cedars-Sinai Medical Center, Los Angeles, CA, USA; 55 Cancer Research Initiatives Foundation, Sime Darby Medical Center, Subang Jaya, Malaysia; 56 Department of Epidemiology, The University of Texas MD Anderson Cancer Center, Houston, TX, USA; 57 Department of Gynaecology, Rigshospitalet, University of Copenhagen, Copenhagen, Denmark; 58 Department of Virus, Lifestyle and Genes, Danish Cancer Society Research Center, Copenhagen, Denmark; 59 Department of Pathology, Molecular Unit, Herlev Hospital, University of Copenhagen, Copenhagen, Denmark; 60 Division of Epidemiology and Prevention, Aichi Cancer Center Research Institute, Nagoya, Aichi, Japan; 61 Department of Statistics, Duke University, Durham, NC, USA; 62 Women’s Cancer Program at the Samuel Oschin Comprehensive, Cancer Institute, Cedars-Sinai Medical Center, Los Angeles, CA, USA; 63 Department of Health Science Research, Division of Biomedical Statistics and Informatics, Mayo Clinic, Rochester, MN, USA; 64 Vesalius Research Center, VIB, University of Leuven, Leuven, Belgium; 65 Department of Oncology, Laboratory for Translational Genetics, University of Leuven, Belgium; 66 Cancer Control Research, BC Cancer Agency, Vancouver, BC, Canada; 67 Department of Cancer Prevention and Control, Roswell Park Cancer Institute, Buffalo, NY, USA; 68 College of Pharmacy and Health Sciences, Texas Southern University, Houston, TX, USA; 69 Department of Obstetrics and Gynaecology, University Malaya Medical Centre, University Malaya, Kuala Lumpur, Malaysia; 70 Department of Cancer Epidemiology and Prevention, M. Sklodowska-Curie Memorial Cancer Center and Institute of Oncology, Warsaw, Poland; 71 Department of Gynecologic Oncology, The University of Texas MD Anderson Cancer Center, Houston, TX, USA; 72 Radboud University Medical Center, Radboud Institute for Molecular Life Sciences, Nijmegen, The Netherlands; 73 Department of Health Research and Policy - Epidemiology, Stanford University School of Medicine, Stanford, CA, USA; 74 Public Health Ontario, Toronto, ON, Canada; 75 Women’s Cancer Research Program, Magee-Women’s Research Institute and University of Pittsburgh Cancer Institute, Pittsburgh, PA, USA; 76 Department of Obstetrics, Gynecology and Reproductive Sciences, University of Pittsburgh School of Medicine, Pittsburgh, PA, USA; 77 Department of Pathology, Rigshospitalet, University of Copenhagen, Copenhagen, Denmark; 78 The University of Texas School of Public Health, Houston, TX, USA; 79 Department of Gynecologic Oncology, Roswell Park Cancer Institute, Buffalo, NY; 80 Department of Epidemiology and Biostatistics, Memorial Sloan-Kettering Cancer Center, New York, NY, USA; 81 Department of Community and Family Medicine, Duke University Medical Center, Durham, NC, USA; 82 Department of Epidemiology, University of Michigan, 1415 Washington Heights, Ann Arbor, Michigan, USA; 83 Channing Division of Network Medicine, Brigham and Women’s Hospital and Harvard Medical School, Boston, MA, USA; 84 Department of Epidemiology, Harvard School of Public Health, Boston, MA, USA; 85 Department of Chronic Disease Epidemiology, Yale School of Public Health, New Haven, CT, USA; 86 Department of Gynecology-Oncology, Princess Margaret Hospital, and Department of Obstetrics and Gynecology, Faculty of Medicine, University of Toronto, Toronto, Ontario, Canada; 87 Institut für Humangenetik, Wiesbaden, Germany; 88 Epidemiology Center and Vanderbilt, Ingram Cancer Center, Vanderbilt University School of Medicine, Nashville, TN, USA; 89 Cancer Epidemiology Program, University of Hawaii Cancer Center, Hawaii, USA; 90 Department of Gynecologic Oncology, Institute of Oncology, Warsaw, Poland; 91 University Malaya Medical Centre, University of Malaya, Kuala Lumpur, Maylaysia; 92 Vanderbilt Epidemiology Center, Vanderbilt University School of Medicine, Nashville, TN, USA; 93 Clinical and Translational Research Organization, All Children’s Hospital Johns Hopkins Medicine, St Petersburg, FL; 94 Department of Obstetrics and Gynecology, Duke University Medical Center, Durham, NC, USA; 95 QIMR Berghofer Medical Research Institute, Brisbane, Australia; 96 Peter MacCallum Cancer Centre, East Melbourne, Australia; 97 Cancer Prevention, Detection & Control Research Program, Duke Cancer Institute, Durham, NC, USA; 98 Department of Public Health Sciences, Medical University of South Carolina, Charleston, SC, USA; 99 Department of Health Science Research, Division of Epidemiology, Mayo Clinic, Rochester, MN, USA; 100 Women’s College Research Institute, University of Toronto, Toronto, Ontario, Canada; 101 The Centre for Cancer Genetic Epidemiology, Department of Oncology, University of Cambridge, Cambridge, UK

## Abstract

Disruption in circadian gene expression, whether due to genetic variation or environmental factors (e.g., light at night, shiftwork), is associated with increased incidence of breast, prostate, gastrointestinal and hematologic cancers and gliomas. Circadian genes are highly expressed in the ovaries where they regulate ovulation; circadian disruption is associated with several ovarian cancer risk factors (e.g., endometriosis). However, no studies have examined variation in germline circadian genes as predictors of ovarian cancer risk and invasiveness. The goal of the current study was to examine single nucleotide polymorphisms (SNPs) in circadian genes *BMAL1, CRY2, CSNK1E, NPAS2, PER3, REV1* and *TIMELESS* and downstream transcription factors *KLF10* and *SENP3* as predictors of risk of epithelial ovarian cancer (EOC) and histopathologic subtypes. The study included a test set of 3,761 EOC cases and 2,722 controls and a validation set of 44,308 samples including 18,174 (10,316 serous) cases and 26,134 controls from 43 studies participating in the Ovarian Cancer Association Consortium (OCAC). Analysis of genotype data from 36 genotyped SNPs and 4600 imputed SNPs indicated that the most significant association was rs117104877 in *BMAL1* (OR = 0.79, 95% CI = 0.68–0.90, p = 5.59 × 10^−4^]. Functional analysis revealed a significant down regulation of *BMAL1* expression following *cMYC* overexpression and increasing transformation in ovarian surface epithelial (OSE) cells as well as alternative splicing of *BMAL1* exons in ovarian and granulosa cells. These results suggest that variation in circadian genes, and specifically *BMAL1*, may be associated with risk of ovarian cancer, likely through disruption of hormonal pathways.

## Introduction

Almost every human cell contains an autonomous circadian clock that synchronizes gene transcription in a daily oscillation for many physiological processes allowing for adaptation to the 24 hour environmental day/night cycle. Circadian genes are known to regulate a variety of cellular processes including the cell cycle, apoptosis, and DNA damage repair [1]. Disruption in circadian gene expression, whether due to genetic variants or environmental factors (e.g., light at night, shiftwork), is associated with increased incidence and invasiveness of a variety of human cancers [2–5] such that in 2007 the International Agency for Research on Cancer classified shift work that involves circadian disruption as “a probable carcinogen” in humans [6]. Disruption of circadian rhythms is also associated with disturbances in menstrual function; female shift workers compared to non-shift workers are more likely to report menstrual irregularity and longer menstrual cycles [7]. Moreover, a recent study found that working nightshifts (i.e., 12:00–4:00 AM) was associated with an increased risk of serious and mucinous, invasive and borderline ovarian tumors in women who were 50 years of age and older [8]. Nevertheless, some studies have failed to find an association between shiftwork and cancer risk [9–11].

The molecular mechanism of the mammalian circadian rhythm is a transcriptional-translational-post-translational autoregulatory feedback loop [12]. The core of the loop consists of CLOCK and BMAL1 proteins, that form a dimer which binds to the E-box region in promoters of period (*PER1, PER2, PER3*) and cryptochrome (*CRY1, CRY2*) genes. Following transcription and translation, PER and CRY proteins form a complex with casein kinase 1 epsilon (CSNK1E) and translocate into the nucleus. Here they bind to BMAL1/CLOCK complex and inhibit their own transcription, which completes the basic auto regulatory loop. PER and CRY proteins are then tagged for proteasomal degradation *via* phosphorylation by CSNK1E and casein kinase 1 delta (CSNK1D) and subsequently by ubiquitination. This cycle lasts approximately 24 h. The BMAL1/CLOCK heterodimer also up regulates the transcription of Rev-erbα and Rora. Their protein products interact with ROR elements (RORE) in the promoter of *BMAL1* gene, upregulating (RORα) or downregulating (REV-ERBα) its transcription [12,13].

Circadian rhythm genes in the hypothalamic suprachiasmatic nucleus (SCN) and reproductive tissues control the timing and length of the ovulatory cycle and pregnancy by their influence on hormones [14]. Estradiol, synthesized in the ovary in response to the stimulation by gonadotropins from the hypothalamic-pituitary-gonadal (HPG) axis, influences the expression of circadian rhythm genes, and in a complex loop-back mechanism the circadian rhythm proteins interfere with estradiol signaling [15]. Overexpression of *CLOCK* transcription factors may play a role in the pathogenesis of endometriosis [16], which is a risk factor for some subtypes of ovarian cancer [17–19]. Infertility is observed in knockout *BMAL1, PER1*, and *PER2* mice [20–22]. These data are consistent with human studies indicating that genetic variation in *BMAL1* is associated with increased rates of miscarriage [23]. Nulliparity is a well-established risk factor for ovarian cancer, although it is currently unclear whether this association is due to infertility or other biological factors (e.g., increased ovulation) [24–27].

Variation in circadian genes has been associated with cancer susceptibility and outcomes. *CLOCK1, CRY1, CRY2, NPAS2, PER1, RORA* and *TIMELESS* variants are associated with breast cancer risk [5,28–33], while polymorphisms in *BMAL1, CLOCK1, CRY1, CRY2, CSNK1E, NPAS2, PER1, PER2*, and *PER3 are* associated with prostate cancer risk [34–36]. *CRY2* and *NPAS2* variation is associated with risk of non-Hodgkin’s lymphoma [37,38] while polymorphisms in *CLOCK1* are associated with colorectal cancer susceptibility [39]. *PER1* and *CLOCK1* variation is associated with glioma risk and outcome [40] and *PER3* polymorphisms have been associated with hepatocellular carcinoma survival [41]. Interestingly, variation in many of these genes is also associated with dysregulation of circadian behaviors, including sleep and activity patterns [42,43], although data are conflicting [44,45]. To date, however, there are no published studies on the association of variation in circadian genes with ovarian cancer risk and invasiveness.

The goal of the current study was to examine variants in seven key circadian rhythm genes (*BMAL1, CRY2, CSNK1E, NPAS2, PER3, REV1, TIMELESS*) and two transcription factors (*KLF10* and *SENP3*) activated by circadian rhythm gene expression as risk factors for epithelial ovarian cancer, histopathologic subtype, and invasiveness. SNPs were evaluated in a two-stage design: a discovery stage using two genome-wide association studies (GWAS) and a replication stage with approximately 44,000 cases and controls from 43 studies that comprise the Ovarian Cancer Association Consortium (OCAC).

## Materials and Methods

### Sample and procedure

The discovery set included 3,761 EOC cases and 2,722 controls in two ovarian cancer GWAS in North America and the United Kingdom (UK). Details of these studies have been previously published [46]. In brief, the North American study was comprised of four case-control studies genotyped using the Illumina 610-quad Beadchip Array™ (i.e., 1,814 cases and 1,867 controls) as well as a single case-control study genotyped on the Illumina 317K and 370K arrays (i.e., 133 cases and 142 controls). The UK study was comprised of four case-only studies genotyped on the Illumina 610-quad Beadchip Array™ and two common control sets genotyped on the Illumina 550K array (i.e., 1,814 cases and 713 controls). The North American and UK studies were analyzed separately and the results combined using fixed effects meta-analysis.

The replication sample consisted of 14,525 invasive EOC cases and 23,447 controls from 43 sites in the Ovarian Cancer Association Consortium (OCAC). An additional 1,747 participants with tumors of low malignant potential were also analyzed. The sample consisted of only participants with European ancestry due to small numbers belonging to other racial groups.

### Gene and SNP selection

Seven essential circadian genes (*BMAL1, CRY2, CSNK1E, NPAS2, PER3, REV1, TIMELESS*) and two key transcription factor genes activated by circadian genes (*KLF10, SENP3*) were selected *a priori* for examination. On the Illumina 610quad, 241 tagSNPs in these genes were identified. The selection of SNPs for replication was informed by ranking of minimal p-values across four sets of results: 1) North American all histologies, 2) North American serous histology, 3) combined GWAS meta-analysis all histologies, and 4) combined GWAS meta-analysis serous histology. Of the 241 SNPs, 37 SNPs were significant in the GWAS discovery set.

### Statistical analysis

Demographic and clinical characteristics of cases and controls were compared using t-tests for continuous variables and chi-square tests for categorical variables. Unconditional logistic regression, treating the number of minor alleles carried as an ordinal variable (i.e., log-additive model), was used to evaluate the association between each SNP and ovarian cancer risk. Per-allele log odds ratios (OR) and their 95% confidence intervals (CI) were estimated. Models were adjusted for study site and population substructure by including study-site indicators and the first five eigenvalues from principal components analysis. The number of principal components was based on the position of the inflexion of the principal components scree plot.

To maximize statistical power, the combined COGS dataset was used to perform SNP-specific analyses for all invasive EOC, the four main histological subtypes (serous, endometrioid, clear cell and mucinous), and tumors of low malignant potential (LMP). Odds ratios specific for each histological subtype were estimated by comparing cases of each subtype to all available controls as reference. Associations with a two-sided p value < 0.05 and a false discovery rate (FDR) q-value [47] < 0.10 were considered to be statistically significant.

### Imputation analyses

These analyses were based on imputed genotypes from the four ovarian cancer GWAS studies (US GWAS, UK GWAS, COGS and Mayo clinic) with a total of 15,398 invasive EOC case subjects and 30,816 control subjects of white-European ancestry. Imputation of each dataset into the 1000 Genomes Project was performed using IMPUTE2 software [48]. We used the 1000 Genomes Project v3 as the reference with pre-phasing of the data using SHAPEIT [49]. SNP log-additive model meta-analysis was carried out for combining results across studies. Only imputed SNPs with r^2^ > 0.25 for each study were used in the analyses.

### Functional analyses

An *in vitro* model of early-stage ovarian cancer has been previously described [45]. Briefly, Illumina HT12 gene expression microarrays were used to profile the transcriptome of 3D models of normal ovarian cells immortalized with *TERT* and overexpressing *cMYC* and a mutant *KRAS* or *BRAF* allele.

## Results

### Sample descriptives

All invasive cancers combined and the four main histological subtypes serous (n = 8,369), endometrioid (n = 2,067), clear cell (n = 1,024) and mucinous (n = 943) were analyzed. Sample characteristics are described in table 1. As expected, significant differences were observed between cases and controls on ovarian cancer risk factors including age, family history of ovarian cancer, age at menarche, body mass index (BMI), history of oral contraceptive use, endometriosis, and number of full term births (p values < 0.05). The proportion of serous histological subtype (57.6%) was higher than the other subtypes (14.2% endometrioid, 7.1% clear cell, 6.5% for mucinous, and 14.6% other).

### Genotyped variants

**Figure 1:**
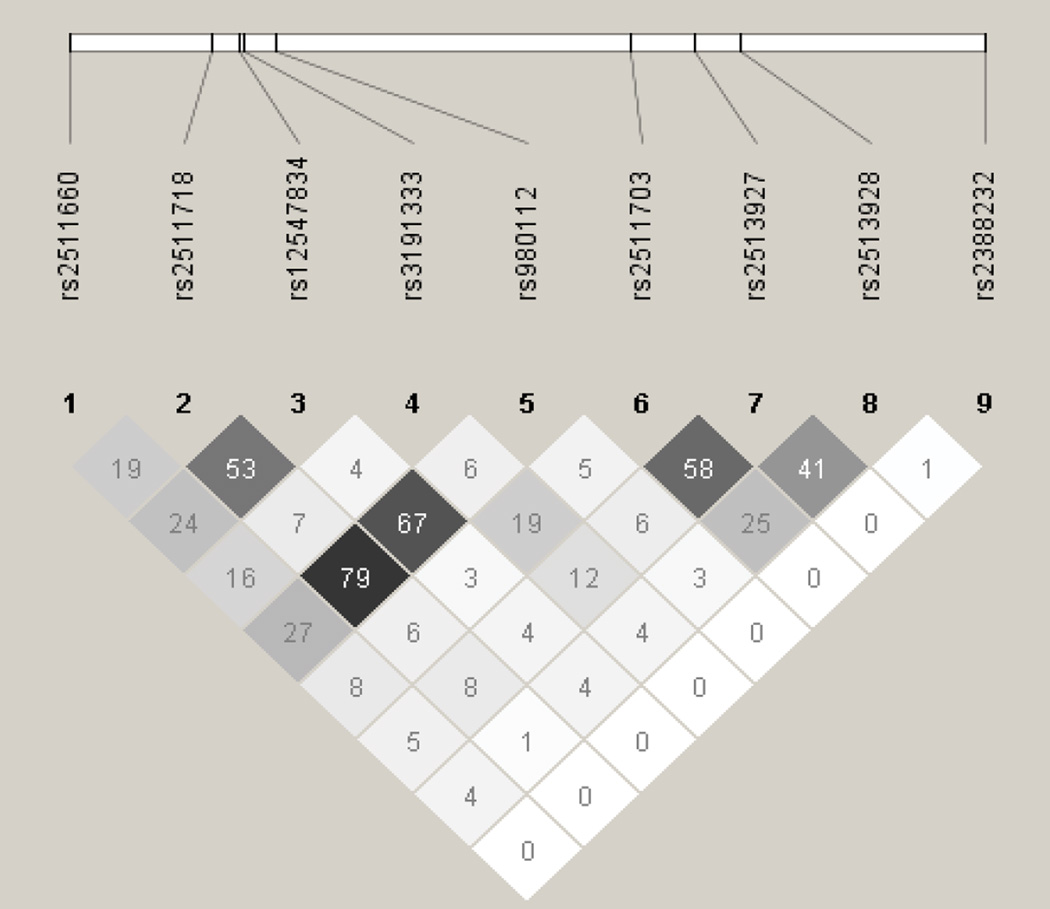
Linkage Disequilibrium (r^2^) among Single Nucleotide Polymorphisms in *KLF10*.

A total of 36 SNPs demonstrated p values < 0.05 in the screening stage and passed quality control. Of these, two in *SENP3* (i.e., rs11656383, rs3499590) were rare variants (i.e., MAFs < 0.01) and were dropped from further analyses. Of the remaining 34 SNPs, 14 were associated with risk of overall EOC, histopathological subtype, and/or invasiveness (Table 2). Seven remained significant after applying the criterion of FDR < 0 .10. Specifically, one SNP was associated with risk of all invasive EOC, rs2513928 in *KLF10* (OR = 0.95, 95% CI = 0.92–0.98, p = 1.75 × 10^−3^). Four SNPs in *KLF10* were associated with risk of serous EOC (rs2513928: OR = 0.94, 95% CI = 0.91–0.98, p = 2.42 × 10^−3^; rs2511703: OR = 1.05, 95% CI = 1.02–1.09, p = 6.54 × 10^−3^; rs3191333: OR = 1.05, 95% CI = 1.02–1.10, p = 6.72 × 10^−3^; rs2513927: OR = 1.05, 95% CI = 1.01–1.09, p = 1.18 × 10^−2^). As shown in figure 1, linkage disequilibrium (LD) between the four significant SNPs in *KLF10* was low to moderate. Risk of endometrioid EOC was associated with *SENP3* rs6608 (OR = 1.13, 95% CI = 1.04–1.23, p = 4.43 ×10^−3^), *CSNK1E* rs135750 (OR = 1.13, 95% CI = 1.03–1.23, p = 7.09 × 10^−3^), *REV1* rs3792152 (OR = 0.92, 95% CI = 0.86–0.98, p = 9.61 × 10^−3^), and *BMAL1* rs10732458 (OR = 1.32, 95% CI = 1.07–1.63, p = 9.64 × 10^−3^). No SNPs were significantly associated with EOC invasiveness nor were any SNPs significantly associated with risk of mucinous or clear cell EOC after applying the criterion of FDR < 0.10.

### Imputed variants

A total of 4600 imputed SNPs in the nine genes of interest (*BMAL1, CRY2, CSNK1E, NPAS2, PER3, REV1, TIMELESS, KLF10, SENP3*) were then examined for association with all invasive EOC. A total of 304 SNPs across all nine genes met criteria for statistical significance (p < 0.05). Top hits in each gene with good imputation quality [r^2^ > 0.8] are shown in table 3. Across all genes, the most significant imputed SNP was rs117104877 in *BMAL1* (OR = 0.79, 95% CI = 0.68–0.90, p = 5.59 × 10^−4^).

### Evaluating the functional role of BMAL1 in ovarian cancer

**Figure 2:**
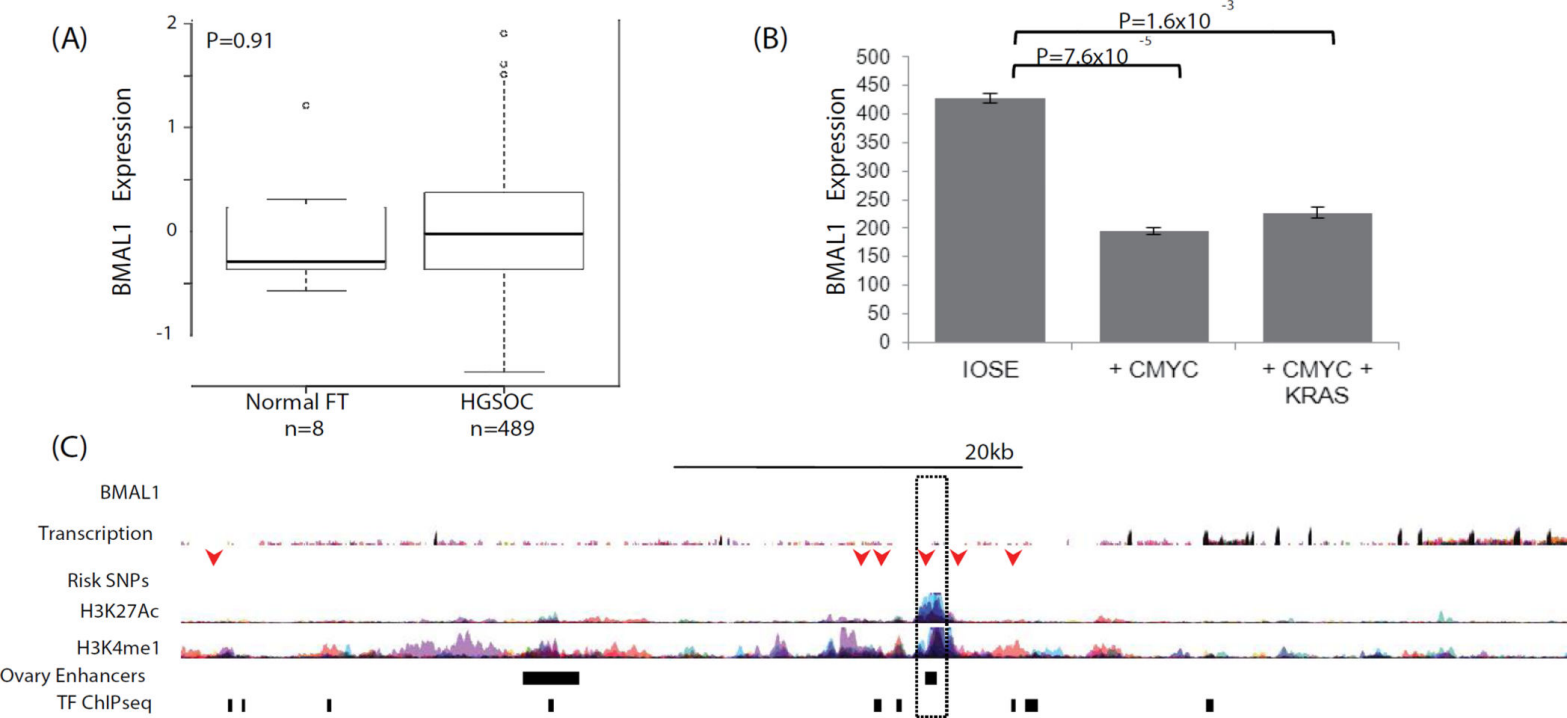
(A) *BMAL1* is not differentially expressed in TCGA expression data for 8 normal fallopian tubes and 489 high-grade serous EOCs; however, in an early stage model of ovarian cancer, (B) *BMAL1* is downregulated in partially transformed ovarian epithelial cells overexpressing *cMYC*. *BMAL1* downregulation is *cMYC* dependent, and not enhanced by the expression of a mutant KRAS allele. (C) 6 SNPs at the *BMAL1* locus coincide with marks of active regulatory elements (H3K27Ac and H3K4me1) or transcription factor binding sites (TF ChiPseq) (arrows). One SNP, rs2896635 coincides with a commonly used enhancer that is active in ovarian stromal tissue (dashed box), and which targets the *BMAL1* gene. ENCODE data and data from [44].

The role of *BMAL1* in ovarian cancer was examined using *in silico* analysis of existing biological datasets in ovarian normal and tumor tissues and an *in vitro* cell biology model of early stage ovarian cancer development. We evaluated gene expression in normal fallopian tubes (n = 8) compared to high-grade serous ovarian carcinomas (HGSOCs, n = 489) using data from The Cancer Genome Atlas (TCGA), but there was no evidence that *BMAL1* was differentially regulated in EOCs as compared to normal tissue (Figure 2).

*BMAL1* expression was further investigated in an early stage transformation model of EOC based on overexpression of *CMYC* in the ovarian surface epithelium (OSE) [50]. *BMAL1* was significantly down regulated in this model, but down regulation was not enhanced by expression of a mutant *KRAS* allele (Figure 2b). Risk associated SNPs were located within intronic regions of *BMAL1* (Figure 2c) and clustered around a commonly described enhancer, suggesting that risk SNPs may influence enhancer activity. Rs2896635 in particular coincides with an enhancer used in many cell types, including an enhancer that is active in ovarian stromal cells that targets the *BMAL1* gene [51]. This suggests that non-cell autonomous signaling pathways may be involved in risk at this locus.

## Discussion

Circadian genes appear to play an important role in regulating reproductive cycles, including ovulation, the length of the estrous cycle, and maintenance of pregnancy. The current study examined variation in nine key genes involved in circadian rhythm regulation or their transcription (*BMAL1, CRY2, CSNK1E, KLF10, NPAS2, PER3, REV1, SENP3, TIMELESS*) as predictors of epithelial ovarian cancer risk, histopathologic subtype, and invasiveness. We found that 14 of the 34 genotyped SNPs in the discovery set were associated with risk of overall EOC, histopathological subtype, and/or invasiveness at p < 0.05. Seven remained significant after applying the criterion of FDR < 0.10. Specifically, risk of overall and serous EOC was associated with variants in *KLF10* while risk of endometrioid EOC was associated with variants in SENP3, *CSNK1E, REV1*, and *BMAL1*. Of 4600 imputed variants in the nine genes of interest, 304 were found to be associated with overall EOC risk at p <. 05. Significant variants were found in all nine genes with the most significant located in *BMAL1*. Additional functional analyses of *BMAL1* indicated that it was down regulated as a consequence of overexpressing cMYC in the OSE, although differential regulation was not observed in HGSOCs compared to normal fallopian tube tissue. Taken together, these results suggest that circadian rhythm genes may play a role in the development of EOC, particularly the genes *KLF10* and *BMAL1*.

While previous research has implicated circadian genes in the development of several types of human cancer, the current study is the first to our knowledge to examine relationships with risk of ovarian cancer. Findings regarding the Krüppel-like factor 10 (*KLF10*) gene are consistent with a sizable body of experimental data indicating that *KLF10* acts to inhibit cellular proliferation and induce apoptosis in a variety of cell types via regulation of transforming growth factor beta (TGFβ) and in turn SMAD [52–58]. *KLF10* is a circadian transcriptional regulator that links the molecular clock to energy metabolism [59]. *KLF10* displays robust BMAL1-dependent circadian expression; the *KLF10* promoter recruits BMAL1 and is transactivated by the CLOCK/BMAL1 dimer through a conserved E-box response element. To our knowledge the role of *KLF10* in human ovarian cancer has not been investigated, although estrogen is known to increase *KLF10* gene transcription [60,61]. *KLF10* expression is reduced in breast tumors relative to normal tissue and is inversely correlated with stage of disease [62,63]. The *KLF10*-*TGFβ*-*SMAD* pathway has been implicated in the development of several other human cancers including those of the prostate, pancreas, kidney, lymphoma, and brain [53,64–67].

Our findings regarding *BMAL1* are interesting in light of data suggesting that this gene may regulate the p53 tumor suppressor pathway. Specifically, silencing of *BMAL1* gene expression prevents cell cycle arrest upon p53 activation in human fibroblast cells [68] and mouse colon and fibroblast cells [69]. These data are consistent with research suggesting that *BMAL1* is transcriptionally silenced via hypermethylation in hematologic malignancies; reintroduction of *BMAL1* causes growth inhibition, while *BMAL1* depletion by RNA interference increases tumor growth [70]. The BMAL1 protein also has been shown to bind to the promoter region of *VEGF* where it regulates transcription and promotes angiogenesis [71].

Evidence suggests that, controlling for stage, histological subtype, and grade, low *BMAL1* and *CRY1* expression together significantly predict lower overall survival in ovarian cancer patients [72]. Previous research also suggests significantly lower *BMAL1* and *CRY1* expression in EOC cells compared to normal ovarian tissue [72]. The current study demonstrated downregulation of *BMAL1* when cMYC was overexpressed in an early stage ovarian cancer transformation model, resulting in increasing ovarian epithelial cell transformation. Nevertheless, we did not observe differential regulation of *BMAL1* when comparing EOC cells to normal fallopian tube tissue. Our findings suggest that down regulation of *BMAL1* may be an early event in ovarian carcinogenesis and that *BMAL1* is a novel cMYC target. SNPs statistically significant in the current study lie within intronic sequences of the *BMAL1* gene and mechanisms by which they impact *BMAL1* expression have yet to be elucidated. Nevertheless, our data suggest that this risk locus may modulate ovarian cancer risk by altering the ovarian stromal microenvironment, for example by influencing the character of ovarian fibroblasts or granulosa cells, both of which express *BMAL1*. In conclusion, our results highlight the significance of circadian rhythm gene variation in EOC susceptibility and suggest an early role for the *BMAL1* gene in EOC pathogenesis.

## Figures and Tables

**Table 1:** Sample demographic and clinical characteristics (n= 37,972).

Characteristics	Controls (n = 23,447) N (%)	Invasive Cases (n = 14,525) N (%)	p-value^2^
Age (years)			
Mean ± SD	55.6 ± 11.9	58.1 ± 11.3	<. 0001
< 40	2027 (8.7)	748 (5.2)	<. 0001
40–49	4771 (20.6)	2544 (17.6)	
50–59	7403 (31.9)	4537 (31.3)	
60–69	6098 (26.3)	4324 (29.8)	
≥ 70	2892 (12.5)	2343 (16.2)	
Family history of ovarian cancer^1^			
No	15425 (92.0)	8634 (82.4)	<. 0001
Yes	1351 (8.0)	1849 (17.6)	
Age at menarche (years)			
Mean ± SD	12.9 ± 1.7	12.8 ± 1.6	0.0314
< 12	3128 (19.3)	1856 (19.2)	0.0772
12	3602 (22.2)	2257 (23.4)	
13	4357 (26.9)	2621 (27.1)	
≥ 14	5112 (31.6)	2923 (30.3)	
Body mass inde × (kg/m^2^)			
< 25	3834 (48.2)	2528 (45.1)	0.0006
25–29	2332 (29.3)	1681 (30.0)	
≥ 30	1797 (22.6)	1396 (24.9)	
Oral contraceptive use			
No	6136 (37.5)	4203 (43.7)	<. 0001
Yes	10230 (62.5)	5419 (56.3)	
Histological subtypes	N/A		
Serous		8369 (57.6)	
Endometroid		2067 (14.2)	
Clear Cell		1024 (7.1)	
Mucinous		943 (6.5)	
Others^3^		2122 (14.6)	

1 for the first degree relatives

2 t-test for a continuous variable and chi-square test for a categorical variable

3 Include mi × ed cell, other specified epithelial, undifferentiated, unknown (but known to be epithelial), nonepithelial, other or unknown if epithelial, or missing

**Table 2:** Associations between Genotyped SNPs in Circadian Genes and EOC Incidence Overall, in Histological Subtypes, and Invasiveness.

					All invasive		Serous		Clear cell	
Gene	SNP	Chr	Min/Maj	MAF	OR (95% CI)	p	OR (95% CI)	p	OR (95% CI)	p
*BMAL1*	rs1026071	11	G/A	0.30	0.98 (0.95–1.01)	2.26 × 10–01	1.00 (0.96–1.04)	9.38 × 10–01	**0.88 (0.8–0.98)**	**1.55 × 10–02**
*BMAL1*	rs10732458	11	A/G	0.02	1.11 (0.99–1.23)	6.91 × 10–02	1.10 (0.96–1.25)	1.64 × 10–01	1.19 (0.88–1.6)	2.52 × 10–01
*BMAL1*	rs10832027	11	G/A	0.33	0.98 (0.95–1.02)	3.48 × 10–01	1.00 (0.96–1.04)	9.79 × 10–01	0.92 (0.84–1.01)	9.15 × 10–02
*BMAL1*	rs1562438	11	A/G	0.29	0.98 (0.95–1.02)	3.07 × 10–01	1.00 (0.96–1.05)	8.46 × 10–01	**0.88 (0.80–0.97)**	**1.35 × 10–02**
*BMAL1*	rs16912751	11	G/A	0.05	0.98 (0.92–1.05)	6.23 × 10–01	0.96 (0.88–1.04)	3.42 × 10–01	1.13 (0.93–1.37)	2.18 × 10–01
*BMAL1*	rs2896635	11	T/A	0.33	0.98 (0.95–1.02)	3.14 × 10–01	1.00 (0.96–1.04)	9.57 × 10–01	0.93 (0.84–1.02)	1.17 × 10–01
*BMAL1*	rs3789327	11	G/A	0.48	1.01 (0.98–1.04)	5.34 × 10–01	1.01 (0.97–1.04)	7.88 × 10–01	1.04 (0.95–1.14)	4.17 × 10–01
*BMAL1*	rs3816360	11	A/G	0.34	1.00 (0.96–1.03)	7.75 × 10–01	1.02 (0.98–1.06)	4.36 × 10–01	**0.91 (0.82–1.00)**	**4.31 × 10–02**
*BMAL1*	rs4757151	11	A/G	0.47	1.00 (0.97–1.04)	7.76 × 10–01	1.01 (0.98–1.05)	5.46 × 10–01	0.97 (0.89–1.06)	5.20 × 10–01
*BMAL1*	rs6486122	11	G/A	0.32	0.98 (0.95–1.02)	2.83 × 10–01	1.00 (0.96–1.04)	9.53 × 10–01	0.92 (0.83–1.01)	8.10 × 10–02
*BMAL1*	rs7117836	11	A/G	0.02	1.10 (0.99–1.22)	8.49 × 10–02	1.09 (0.96–1.24)	1.65 × 10–01	1.19 (0.89–1.59)	2.46 × 10–01
*BMAL1*	rs7947951	11	A/G	0.32	0.99 (0.95–1.02)	3.60 × 10–01	1.00 (0.96–1.04)	9.13 × 10–01	0.92 (0.84–1.01)	9.30 × 10–02
*CRY2*	rs11038695	11	A/G	0.08	1.05 (0.99–1.11)	1.11 × 10–01	1.03 (0.97–1.11)	3.40 × 10–01	0.99 (0.84–1.17)	9.25 × 10–01
*CSNK1E*	rs135750	22	G/C	0.15	1.04 (1.00–1.09)	6.14 × 10–02	1.03 (0.98–1.08)	3.12 × 10–01	1.00 (0.89–1.13)	9.73 × 10–01
*KLF10*	rs12547834	8	G/A	0.07	0.96 (0.90–1.02)	1.43 × 10–01	0.94 (0.88–1.02)	1.20 × 10–01	1.02 (0.85–1.21)	8.49 × 10–01
*KLF10*	rs3191333	8	A/G	0.37	**1.04 (1.01–1.07)**	**2.42 × 10–02**	**1.05 (1.02–1.10)**	**6.72 × 10–03**	1.04 (0.95–1.14)	3.95 × 10–01
*KLF10*	rs980112	8	A/G	0.10	0.97 (0.92–1.02)	1.98 × 10–01	0.96 (0.90–1.03)	2.42 × 10–01	1.06 (0.92–1.23)	4.08 × 10–01
*KLF10*	rs2388232	8	G/A	0.27	1.01 (0.97–1.04)	7.92 × 10–01	1.00 (0.96–1.04)	9.22 × 10–01	**1.11 (1.01–1.23)**	**2.91 × 10–02**
*KLF10*	rs2511703	8	G/A	0.43	**1.04 (1.01–1.07)**	**1.83 × 10–02**	**1.05 (1.02–1.09)**	**6.54 × 10–03**	1.00 (0.91–1.09)	9.55 × 10–01
*KLF10*	rs2513927	8	A/G	0.49	**1.04 (1.01–1.07)**	**1.86 × 10–02**	**1.05 (1.01–1.09)**	**1.18 × 10–02**	1.00 (0.91–1.10)	9.79 × 10–01
*KLF10*	rs2513928	8	G/A	0.46	**0.95 (0.92–0.98)**	**1.75 × 10–03**	**0.94 (0.91–0.98)**	**2.42 × 10–03**	0.94 (0.85–1.02)	1.50 × 10–01
*KLF10*	rs2511660	8	A/G	0.22	0.97 (0.94–1.01)	1.57 × 10–01	0.96 (0.92–1.00)	6.95 × 10–02	0.99 (0.89–1.10)	8.56 × 10–01
*KLF10*	rs2511718	8	A/G	0.12	0.98 (0.94–1.03)	4.57 × 10–01	0.98 (0.92–1.04)	4.47 × 10–01	1.06 (0.93–1.22)	3.68 × 10–01
*NPAS2*	rs1053091	2	A/G	0.02	1.05 (0.93–1.19)	4.14 × 10–01	1.10 (0.96–1.27)	1.83 × 10–01	1.12 (0.79–1.59)	5.17 × 10–01
*NPAS2*	rs13012930	2	A/G	0.17	**0.96 (0.92–1.00)**	**4.80 × 10–02**	**0.95 (0.91–1.00)**	**4.11 × 10–02**	0.98 (0.87–1.10)	6.86 × 10–01
*NPAS2*	rs3768988	2	G/A	0.06	1.01 (0.95–1.07)	8.18 × 10–01	1.02 (0.94–1.10)	6.44 × 10–01	1.01 (0.84–1.22)	9.09 × 10–01
*NPAS2*	rs7573323	2	A/G	0.03	0.97 (0.88–1.07)	5.47 × 10–01	0.99 (0.88–1.11)	8.61 × 10–01	0.87 (0.65–1.18)	3.73 × 10–01
*PER3*	rs228644	1	A/G	0.40	1.00 (0.97–1.03)	9.23 × 10–01	1.00 (0.96–1.03)	8.38 × 10–01	0.97 (0.89–1.07)	5.45 × 10–01
*PER3*	rs228682	1	G/A	0.40	1.00 (0.97–1.03)	7.83 × 10–01	0.99 (0.96–1.03)	7.32 × 10–01	0.97 (0.88–1.06)	4.84 × 10–01
*PER3*	rs228698	1	A/G	0.04	1.00 (0.93–1.08)	9.73 × 10–01	0.99 (0.90–1.08)	7.67 × 10–01	0.90 (0.71–1.14)	3.79 × 10–01
*PER3*	rs697693	1	A/G	0.19	0.99 (0.95–1.03)	5.55 × 10–01	0.98 (0.94–1.03)	5.02 × 10–01	1.07 (0.96–1.19)	2.46 × 10–01
*REV1*	rs3792152	2	A/G	0.44	0.97 (0.94–1.00)	6.47 × 10–02	0.97 (0.94–1.01)	1.34 × 10–01	0.99 (0.90–1.08)	7.96 × 10–01
*SENP3*	rs6608	17	A/G	0.17	**1.05 (1.00–1.09)**	**3.35 × 10–02**	1.04 (0.99–1.09)	1.42 × 10–01	1.01 (0.90–1.14)	8.81 × 10–01
*TIMELES*	*S* rs7302060	12	G/A	0.41	0.99 (0.96–1.02)	3.53 × 10–01	0.98 (0.94–1.01)	2.09 × 10–01	0.97 (0.88–1.06)	4.77 × 10–01

		Endometriod		Mucinous		LMP vs. controls		Invasive vs. LMP	
Gene	SNP	OR (95% CI)	p	OR (95% CI)	p	OR (95% CI)	p	OR (95% CI)	p
*BMAL1*	rs1026071	0.98 (0.91–1.05)	5.17 × 10–01	0.94 (0.85–1.05)	2.63 × 10–01	0.99 (0.92–1.07)	8.95 × 10–01	1.00 (0.92–1.08)	9.17 × 10–01
*BMAL1*	rs10732458	**1.32 (1.07–1.63)**	**9.64 × 10–03**	1.02 (0.72–1.44)	9.12 × 10–01	0.77 (0.58–1.02)	6.51 × 10–02	**1.44 (1.09–1.92)**	**1.17 × 10–02**
*BMAL1*	rs10832027	0.99 (0.93–1.06)	8.48 × 10–01	0.95 (0.86–1.05)	2.75 × 10–01	1.00 (0.93–1.07)	9.17 × 10–01	1.00 (0.92–1.07)	9.04 × 10–01
*BMAL1*	rs1562438	0.97 (0.90–1.04)	4.12 × 10–01	0.94 (0.85–1.05)	2.74 × 10–01	1.00 (0.93–1.08)	9.79 × 10–01	0.99 (0.92–1.07)	8.80 × 10–01
*BMAL1*	rs16912751	0.90 (0.78–1.05)	1.97 × 10–01	1.11 (0.91–1.36)	2.94 × 10–01	0.88 (0.75–1.04)	1.40 × 10–01	1.12 (0.95–1.33)	1.73 × 10–01
*BMAL1*	rs2896635	0.99 (0.92–1.06)	7.20 × 10–01	0.95 (0.86–1.05)	3.04 × 10–01	1.00 (0.93–1.07)	9.49 × 10–01	0.99 (0.92–1.07)	8.21 × 10–01
*BMAL1*	rs3789327	1.00 (0.94–1.07)	9.84 × 10–01	0.95 (0.86–1.04)	2.53 × 10–01	1.01 (0.94–1.08)	8.63 × 10–01	1.01 (0.94–1.08)	8.01 × 10–01
*BMAL1*	rs3816360	0.99 (0.92–1.06)	7.74 × 10–01	0.94 (0.85–1.04)	2.52 × 10–01	1.02 (0.94–1.09)	6.67 × 10–01	0.99 (0.92–1.06)	7.53 × 10–01
*BMAL1*	rs4757151	0.99 (0.92–1.05)	6.91 × 10–01	1.06 (0.97–1.17)	1.97 × 10–01	0.98 (0.91–1.05)	5.61 × 10–01	1.03 (0.96–1.11)	3.73 × 10–01
*BMAL1*	rs6486122	0.99 (0.92–1.06)	6.90 × 10–01	0.95 (0.86–1.05)	3.12 × 10–01	0.99 (0.92–1.07)	8.62 × 10–01	0.99 (0.92–1.07)	8.83 × 10–01
*BMAL1*	rs7117836	**1.24 (1.01–1.54)**	**4.40 × 10–02**	1.06 (0.76–1.48)	7.36 × 10–01	**0.76 (0.57–1.00)**	**4.82 × 10–02**	**1.45 (1.09–1.92)**	**9.81 × 10–03**
*BMAL1*	rs7947951	0.99 (0.93–1.06)	8.17 × 10–01	0.95 (0.86–1.05)	2.94 × 10–01	1.00 (0.93–1.07)	9.34 × 10–01	0.99 (0.92–1.07)	8.78 × 10–01
*CRY2*	rs11038695	1.09 (0.97–1.22)	1.48 × 10–01	0.97 (0.82–1.15)	7.19 × 10–01	1.07 (0.94–1.21)	2.88 × 10–01	0.98 (0.86–1.11)	7.02 × 10–01
*CSNK1E*	rs135750	**1.13 (1.03–1.23)**	**7.09 × 10–03**	1.06 (0.93–1.20)	3.90 × 10–01	1.02 (0.93–1.12)	6.98 × 10–01	1.03 (0.93–1.13)	6.10 × 10–01
*KLF10*	rs12547834	0.99 (0.87–1.13)	8.75 × 10–01	0.86 (0.71–1.04)	1.22 × 10–01	0.97 (0.84–1.11)	6.56 × 10–01	0.99 (0.86–1.14)	8.87 × 10–01
*KLF10*	rs3191333	1.03 (0.96–1.10)	3.84 × 10–01	0.95 (0.86–1.05)	3.01 × 10–01	0.99 (0.92–1.06)	7.70 × 10–01	1.05 (0.97–1.13)	2.21 × 10–01
*KLF10*	rs980112	0.95 (0.85–1.06)	3.49 × 10–01	0.90 (0.76–1.05)	1.85 × 10–01	0.99 (0.88–1.12)	8.95 × 10–01	0.98 (0.87–1.11)	7.73 × 10–01
*KLF10*	rs2388232	0.99 (0.92–1.06)	7.81 × 10–01	0.98 (0.88–1.08)	6.40 × 10–01	1.03 (0.96–1.12)	3.86 × 10–01	0.97 (0.90–1.05)	4.71 × 10–01
*KLF10*	rs2511703	1.05 (0.98–1.12)	1.34 × 10–01	0.95 (0.87–1.05)	3.17 × 10–01	0.98 (0.91–1.05)	5.75 × 10–01	1.06 (0.98–1.13)	1.32 × 10–01
*KLF10*	rs2513927	1.05 (0.99–1.13)	1.11 × 10–01	0.94 (0.85–1.03)	1.71 × 10–01	0.98 (0.92–1.05)	5.94 × 10–01	1.06 (0.99–1.13)	1.24 × 10–01
*KLF10*	rs2513928	0.95 (0.89–1.01)	1.20 × 10–01	1.02 (0.93–1.12)	6.88 × 10–01	0.96 (0.90–1.03)	2.56 × 10–01	0.99 (0.92–1.06)	6.95 × 10–01
*KLF10*	rs2511660	1.02 (0.94–1.10)	6.80 × 10–01	0.96 (0.85–1.07)	4.38 × 10–01	1.06 (0.98–1.15)	1.43 × 10–01	**0.92 (0.85–1.00)**	**4.47 × 10–02**
*KLF10*	rs2511718	0.96 (0.87–1.06)	4.28 × 10–01	0.95 (0.82–1.09)	4.52 × 10–01	1.01 (0.91–1.13)	8.02 × 10–01	0.97 (0.87–1.09)	6.41 × 10–01
*NPAS2*	rs1053091	0.84 (0.64–1.12)	2.45 × 10–01	1.02 (0.71–1.47)	9.00 × 10–01	1.10 (0.85–1.44)	4.69 × 10–01	0.93 (0.71–1.22)	5.88 × 10–01
*NPAS2*	rs13012930	1.02 (0.93–1.11)	7.23 × 10–01	0.91 (0.80–1.03)	1.31 × 10–01	1.04 (0.95–1.14)	3.73 × 10–01	0.92 (0.84–1.01)	9.47 × 10–02
*NPAS2*	rs3768988	0.93 (0.81–1.07)	3.02 × 10–01	1.01 (0.84–1.22)	8.90 × 10–01	1.09 (0.95–1.26)	2.06 × 10–01	0.93 (0.80–1.07)	2.83 × 10–01
*NPAS2*	rs7573323	0.92 (0.74–1.13)	4.13 × 10–01	0.83 (0.60–1.16)	2.78 × 10–01	0.83 (0.66–1.05)	1.12 × 10–01	1.18 (0.93–1.49)	1.76 × 10–01
*PER3*	rs228644	0.97 (0.91–1.04)	3.76 × 10–01	1.07 (0.97–1.17)	1.82 × 10–01	0.99 (0.92–1.06)	6.91 × 10–01	1.02 (0.95–1.09)	6.69 × 10–01
*PER3*	rs228682	0.97 (0.91–1.04)	3.51 × 10–01	1.07 (0.97–1.17)	1.90 × 10–01	0.98 (0.92–1.06)	6.37 × 10–01	1.02 (0.95–1.09)	6.65 × 10–01
*PER3*	rs228698	1.04 (0.89–1.23)	6.04 × 10–01	1.08 (0.86–1.36)	4.89 × 10–01	0.96 (0.81–1.15)	6.66 × 10–01	1.01 (0.85–1.21)	8.76 × 10–01
*PER3*	rs697693	0.99 (0.91–1.08)	8.61 × 10–01	0.92 (0.81–1.04)	1.67 × 10–01	1.04 (0.95–1.13)	3.81 × 10–01	0.96 (0.88–1.05)	3.45 × 10–01
*REV1*	rs3792152	**0.92 (0.86–0.98)**	**9.61 × 10–03**	0.99 (0.90–1.09)	8.32 × 10–01	0.98 (0.91–1.05)	4.87 × 10–01	1.01 (0.94–1.09)	7.65 × 10–01
*SENP3*	rs6608	**1.13 (1.04–1.23)**	**4.43 × 10–03**	1.00 (0.88–1.14)	9.90 × 10–01	1.01 (0.92–1.10)	9.00 × 10–01	1.04 (0.94–1.14)	4.79 × 10–01
*TIMELESS*	rs7302060	1.01 (0.95–1.08)	7.22 × 10–01	0.97 (0.88–1.07)	5.10 × 10–01	**0.93 (0.87–1.00)**	**4.86 × 10–02**	1.06 (0.99–1.14)	1.09 × 10–01

SNP: Single Nucleotide Polymorphism, Chr: Chromosome, Min/Maj: Minor and Major Allele, MAF: Minor Allele Frequency, LMP: Low Malignant Potential, OR: Odds Ratio

**Note:** odds ratio is calculated based on per-minor allele, bolded SNPs indicate an association of p < 0.05 with overall EOC or histologic subtype.

**Table 3:** Associations between the Top Imputed SNP in Each Gene with Good Imputation Quality (r^2^ > 0.8) and EOC Incidence Overall.

Gene	SNP	Min/Maj	MAF	OR (95% CI)	p
***BMAL1***	rs117104877	G/A	0.017	0.79 (0.68–0.90)	5.59 × 10–4
***CRY2***	rs10838527	G/A	0.082	1.05 (0.99–1.11)	7.66 × 10–2
***CSNK1E***	rs111427515	G/T	0.008	1.25 (1.06–1.47)	6.60 × 10–3
***KLF10***	rs2511699	A/G	0.461	0.96 (0.93–0.99)	4.13 × 10–3
***NPAS2***	rs732375	T/A	0.134	1.07 (1.02–1.11)	3.76 × 10–3
***PER3***	rs228640	A/G	0.297	1.04 (1.01–1.07)	1.24 × 10–2
***REV1***	rs3792146	T/C	0.547	1.03 (1–1.06)	2.71 × 10–2
***SENP3***	rs143094271	A/G	0.023	0.86 (0.77–0.95)	4.01 × 10–3
***TIMELESS***	rs2638286	C/T	0.030	1.05 (0.96–1.15)	2.56 × 10–1

SNP: Single Nucleotide Polymorphism, Min/Maj: Minor and Major Allele, MAF: Minor Allele Frequency, OR: Odds Ratio

**Note:** odds ratio is calculated based on per-minor allele
